# Gut physiology of rainbow trout (*Oncorhynchus mykiss*) is influenced more by short-term fasting followed by refeeding than by feeding fishmeal-free diets

**DOI:** 10.1007/s10695-024-01339-0

**Published:** 2024-04-16

**Authors:** Laura Frohn, Diogo Peixoto, Frédéric Terrier, Benjamin Costas, Jérôme Bugeon, Christel Cartier, Nadège Richard, Karine Pinel, Sandrine Skiba-Cassy

**Affiliations:** 1 INRAE, NUMEA, Université de Pau & des Pays de l’Adour, E2S UPPA, 64310 Saint Pée-sur-Nivelle, France; 2 Phileo By Lesaffre, 59700 Marcq-en-Barœul, France; 3 ICBAS - Instituto de Ciências Biomédicas Abel Salazar, Universidade Do Porto, 4050-313 Porto, Portugal; 4 CIIMAR - Centro Interdisciplinar de Investigação Marinha E Ambiental, 4450-208 Matosinhos, Portugal; 5 INRAE, LPGP, Fish Physiology and Genomics, 35000 Rennes, France; 6 INRAE, ToxAlim, ENVT, INP El Purpan, UPS, 31027 Toulouse, France

**Keywords:** Rainbow trout, Intestine, Immunity, Inflammation, Fasting

## Abstract

Supplementing a fishmeal-free diet with yeast extract improves rainbow trout (*Oncorhynchus mykiss*) growth performance and modulates the hepatic and intestinal transcriptomic response. These effects are often observed in the long term but are not well documented after short periods of fasting. Fasting for a few days is a common practice in fish farming, especially before handling the fish, such as for short sorting, tank transfers, and vaccinations. In the present study, rainbow trout were subjected to a 4-day fast and then refed, for 8 days, a conventional diet containing fishmeal (control diet) or alternative diets composed of terrestrial animal by-products supplemented or not with a yeast extract. During the refeeding period alone, most of the parameters considered did not differ significantly in response to the different feeds. Only the expression of claudin-15 was upregulated in fish fed the yeast-supplemented diet compared to the control diet. Conversely, fasting followed by refeeding significantly influenced most of the parameters analyzed. In the proximal intestine, the surface area of villi significantly increased, and the density of goblet cell tended to decrease during refeeding. Although no distinct plasma immune response or major signs of gut inflammation were observed, some genes involved in the structure, complement pathway, antiviral functions, coagulation, and endoplasmic reticulum stress response of the liver and intestine were significantly regulated by refeeding after fasting. These results indicate that short-term fasting, as commonly practiced in fish farming, significantly alters the physiology of the liver and intestine regardless of the composition of the diet.

**Supplementary Information:**

The online version contains supplementary material available at 10.1007/s10695-024-01339-0.

## Introduction

Aquaculture-rearing practices and feeds changed greatly during the twentieth century. Development of extruded pellets in the late 1980s and the gradual replacement of fishmeal and fish oil with plant ingredients (e.g., whole grains, oilseeds, legumes) helped address the constraints faced by the sector, particularly in reducing human pressures on wild forage-fish populations (Tacon and Metian 2009; Bandara 2018). From 1990 to 2020, especially for Atlantic salmon (*Salmo salar*), the contents of fish oil and fishmeal in feed decreased from 65.4% and 24.0% to 12.1% and 10.3%, respectively, with no influence on zootechnical performances (Aas et al. 2019). Nonetheless, most carnivorous fish species, such as salmonids (salmon and trout), are sensitive to dietary changes, which prohibit completely replacing fishmeal and fish oil in their feed. Thus, for these species, plant-based diets are likely to influence feed intake, nutrient digestion, absorption, and assimilation (e.g., metabolism) (Bureau et al. 1998; Panserat et al. 2009; Li et al. 2014). These diets can also degrade intestinal health by causing inflammation in the digestive tract, likely due to the presence of anti-nutritional factors (e.g., oligosaccharides, lectins, saponins) in the plant ingredients (Francis et al. 2001; Gatlin et al. 2007). Therefore, plant-based diets are likely to decrease the growth and degrade the health of the fish, thus rendering them more sensitive to stress (Krogdahl et al. 2010; Lazzarotto et al. 2015).

In addition to plant ingredients, the aquaculture sector is considering other replacements for marine ingredients, such as insect meals, microalgae, and industrial by-products from terrestrial animals. However, their use is restricted due to variable growth performances that result from their inconsistent quality and nutritional values, as well as the amounts included in diets (Galkanda‐Arachchige et al. 2020). Including functional ingredients can meet the objectives of maintaining acceptable growth performances and mitigating adverse effects of alternative diets. For example, yeast can be included in whole or in derivative form (i.e., cell wall, cytosolic fraction, purified components). Including yeast in feed has been shown to provide many benefits for most terrestrial and aquatic livestock, such as improvements in feed palatability, nutrient digestibility (due to enzyme production and intestinal pH regulation), the immune system and pathogen resistance (due to bioactive components such as MOS, β-glucan, nucleotides, and peptides), the quality of meat and milk, egg production of poultry, and growth performances of fish and pigs (Gatesoupe 2007; Vohra et al. 2016; Agboola et al. 2021). The performance of new feeds or the benefits of feed additives are often examined over the long term to primarily assess effects on growth without necessarily analyzing the early physiological responses of animals. When using a new diet, long-term responses could be the result of mechanisms that fish gradually implement to adapt to change in formulation. Short-term responses, on the other hand, could reflect an acute homeostatic response involving the mobilization of mechanisms aimed at restoring or maintaining metabolic balance in a perturbed environment, in this case a change in diet. Indeed, a study of Atlantic salmon fed a soybean meal diet showed that diet can have effects on the first day of exposure, particularly on intestinal histopathology and on the regulation of genes involved in immunity, detoxification, cellular repair, and metabolism (Sahlmann et al. 2013).

Short periods of fasting followed by refeeding are frequently used in aquaculture. On fish farms, short-term fasting is strongly recommended and commonly applied during periods of stress (i.e., animal handling, transport between different production sites, epizootic situations) or in order to induce compensatory growth (Dobson and Holmes 1984; Cho 2005; Jena et al. 2017). During short-term fasting (less than 2 weeks), the metabolic rate of fish is lowered and causes the energy required for digestion to be directed to vital functions such as maintaining cellular homeostasis, brain function, and respiration, without necessarily causing significant weight loss (Caruso et al. 2012; Karatas et al. 2021). Like birds and mammals, fish in their natural environment are also frequently subjected to long periods of fasting or starvation (greater than 2 weeks) during winter, migration, and reproduction events (Navarro and Gutiérrez 1995; Bar 2014). Indeed, even when short, these periods influence gut microbiota (Xia et al. 2014), metabolism (Black and Skinner 1986), and digestive and hepatic physiology (Martin et al. 2001; Krogdahl and Marie Bakke-McKellep 2005; Bar 2014).

Utilization of alternative diets without fishmeal during 12 weeks impacted the growth performance of juvenile rainbow trout (*Oncorhynchus mykiss*) and these phenotypes were accompanied by changes in hepatic and intestinal transcriptomic profiles, as well as structural changes in the intestine (Frohn et al. 2024). As no short-term evaluation has been carried out on this type of feed, we first compared the early effect within the first 8 days of feeding a commercial-like diet containing fishmeal and fish oil, an alternative diet based on plant ingredients and terrestrial animal by-products which had been shown to reduce the growth performance of juvenile rainbow trout (*Oncorhynchus mykiss*), and the same diet supplemented with yeast extract which significantly improved growth (Frohn et al. 2024). Like the use of a novel feed, we then hypothesized that refeeding after a short fasting period could also influence the fish’s early physiological response, which is rarely accounted for, despite the frequent implementation of fasting and refeeding periods in fish farms. Consequently, this study also aimed to evaluate the effects of a short 4-day fasting period followed by refeeding with the above-mentioned feeds on intestinal histology and parameters related to defense and protection mechanisms in juvenile rainbow trout.

## Materials and methods

### Feed formulation and experimental design

Three experimental feeds were formulated to meet the nutritional requirements of the rainbow trout according to NRC recommendations (Council NR 2011). The control diet (CTL) was formulated as close as possible to a commercial feed for rainbow trout and contained 19% fishmeal and 7.0% fish oil. Two processed animal protein diets (10% dehydrated poultry protein, 6% hydrolyzed feather meal, and 1% poultry and pig blood meal) were also formulated: one not supplemented (PAP) and one supplemented with yeast extract (PAP + YE). The yeast extract used contained the cytosolic fraction of the yeast *Saccharomyces cerevisiae* (Prosaf®, Phileo by Lesaffre, Marcq-en-Barœul, France)*.* The contents of other ingredients in the experimental feeds varied (Table 1).

**Table 1:** Ingredients and proximate composition of three experimental diets: commercial-like feed (CTL), processed animal protein feed (PAP), and PAP with 3% yeast extract (PAP + YE)

	CTL	PAP	PAP + YE
*Ingredient (%)*			
Corn gluten	15.00	17.90	16.00
Soy protein concentrate	14.03	11.00	12.00
Soybean meal	12.00	10.52	8.34
Faba bean	8.00	12.00	8.00
Whole wheat	10.76	9.22	13.01
Fishmeal	19.00	-	-
Dehydrated poultry protein	-	10.00	10.00
Hydrolyzed feather meal	-	6.00	6.00
Poultry and pig blood meal	-	1.00	1.00
Rapeseed oil	12.18	10.45	10.54
Fish oil	7.03	7.87	7.91
Yeast extract^1^	-	-	3.00
L-lysine	-	0.27	0.49
L-methionine	-	0.26	0.29
Soy lecithin	-	0.51	0.42
Vitamin premix^2^	1.00	1.00	1.00
Dicalcium phosphate	-	1.00	1.00
Mineral premix^3^	1.00	1.00	1.00
*Proximate composition*^*4*^			
Dry matter (DM) (%)	97.1	97.4	97.2
Protein (%DM)	46.9	46.5	46.3
Lipid (%DM)	22.1	21.6	20.8
Energy (%DM)	25.0	25.1	24.9
Ash (%DM)	6.8	5.5	5.6
Starch (%DM)	12.1	13.5	14.2

^1^Cytosolic fraction of *Saccharomyces cerevisiae* (Prosaf®, Phileo by Lesaffre, Marcq-en-Barœul, France)

^2^Provided per 100 g of premix: vitamin A 500,000 IU, vitamin D_3_ 250,000 IU, vitamin E 500 mg, vitamin C 1429 mg, vitamin B_1_ 10 mg, vitamin B_2_ 50 mg, vitamin B_3_ 100 mg, vitamin B_5_ 200 mg, vitamin B_6_ 30 mg, vitamin B_7_ 3000 mg, vitamin B_8_ 100 mg, vitamin B_9_ 10 mg, vitamin B_12_, 100 mg, vitamin K_3_ 200 mg, folic acid 10 mg, biotin 100 mg, choline chloride 16,700 mg, and cellulose 76,921 mg

^3^Provided per 100 g of premix: calcium hydrogen phosphate 49,478 mg, calcium carbonate 21,500 mg, sodium chloride 4000 mg, potassium chloride 9000 mg, magnesium oxide 12,400 mg, iron sulfate 2000 mg, zinc sulfate 900 mg, manganese sulfate 300 mg, copper sulfate 300 mg, cobalt chloride 2 mg, potassium iodide 15 mg, sodium selenite 5 mg, and sodium fluoride 100 mg

^4^Analyzed values

A schematic diagram of the experimental protocol is shown in Supplementary Fig. 1. One hundred and twenty (120) rainbow trout, with an average biomass of 232 ± 39 g, were distributed in four 100 L tanks in groups of 30 fish (initial density: 55 kg/m^3^). Tanks were continuously supplied with well-oxygenated freshwater at 17 °C (flow-through system), with concentrations of 9.4 mg/L at the inlet and 7.5 mg/L at the outlet of the tank. After batching, the fish were acclimated to their new environment and fed a commercial feed (T3P Omega®, Skretting, Fontaine-les-Vervins, France) for 3 days (distributed daily to visual satiation) and then fasted for 4 days. The duration of this fasting period was applied in accordance with estimated gastric emptying rates in rainbow trout and Atlantic salmon (Windell et al. 1976; Aas et al. 2017). Compared to these studies, the duration was slightly increased to ensure that the gastrointestinal tracts were completely empty, allowing only the effect of the experimental diets to be considered during the refeeding period. To evaluate the effects of fasting followed by refeeding, fish from the first tank were not refed after fasting and were used to represent day 0 and the “fasted fish” group of the experiment. In this tank, six individuals were anesthetized and then euthanized in baths containing 50 and 150 mg tricaine/L of water, respectively. Then, blood was drawn from the caudal vein using a 2.5 mL EDTA-treated syringe and a 0.6 × 32 mm needle, centrifuged at 3000 g for 10 min (microcentrifuge 1–15 P, Sigma Centrifuges, Osterode am Harz, Germany), and the plasma extracted was stored at − 20 °C until plasma immune markers were quantified. Whole liver samples and intestine (proximal and distal parts) were collected, soaked in RNA later and immediately immersed in liquid nitrogen and stored at − 80 °C until molecular analyses were performed. Additional samples of the proximal and distal intestine were fixed in a 10% buffered formalin solution before being processed for histological analysis. In order to assess the early effect of novel diets, the other 3 tanks (tanks 2 to 4) were randomly assigned to one of the three experimental feeds (Table 1), and fish were refed twice a day until visual satiation over an 8-day period, selected based on the study by Sahlmann et al. (2013). On days 2, 5, and 8 of refeeding, 6 fish per tank were anesthetized, euthanized, and sampled as described above, 6 h after the last meal, as previously done by Richard et al. (2021). Final density on day 8 was 23 kg/m^3^.

### Plasma immune markers

Activities of the alternative complement pathway, lysozyme, and peroxidase were measured in the plasma, as described by Frohn et al. (2024). Total antiprotease activity was determined by assessing the ability of plasma to inhibit trypsin activity, as described by Peixoto et al. (2022).

### Histology of intestine

According to the recommendations of Feldman and Wolfe (2014), samples of the proximal and distal intestine were fixed in buffered formalin, dehydrated in successive ethanol baths, clarified in xylene, and embedded in paraffin blocks. Transverse intestine sections 2 µm thick were cut using a semi-automatic rotating microtome (HM 340E, Microm Microtech, Brignais, France). Tissues were then stained with periodic acid–Schiff alcian blue to assess gut morphology and count the number of goblet cells in the intestinal mucosa, as it has been described by Frohn et al. (2024).

### Image analysis

**Fig. 1:**
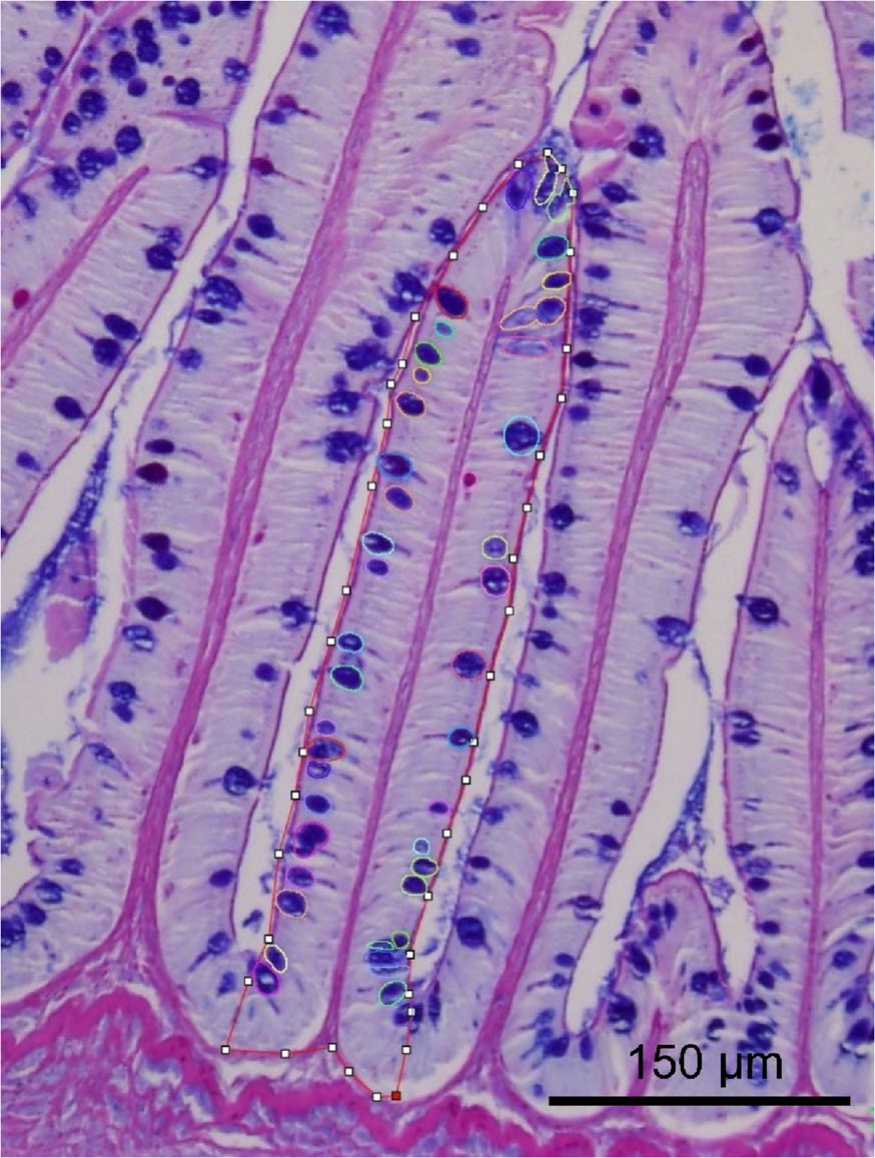
Example of manual delineation of a villus (red line) and detection of goblet cells (circles) using FIJI software

Images of the intestinal mucosa were obtained using a microscope (DMRB, Leica, Wetzlar, Germany) equipped with a digital camera (DP71 1.4 M pixels, Olympus, Tokyo, Japan). The images were then processed using two macros that were created using the MorphoLibJ (Legland et al. 2016) and StarDist (Schmidt et al. 2018) plugins of FIJI 1.53t software (Schindelin et al. 2012). The first macro measured the surface area (µm^2^) of a given villus and counted the number of goblet cells in it, in order to calculate the density of goblet cells (per mm^2^) (Fig. 1). The cells were detected using the StarDist Versatile (H&E nuclei) model, and each villus of interest was manually delineated (polygon-selection tool). The cells inside the villus were manually corrected if incorrectly segmented. The second macro measured villi height (µm) manually using the segmented-line tool of FIJI. Five villi per section were analyzed.

### Molecular analysis: RNA extraction and real-time RT-qPCR

Total RNA was extracted from the liver, proximal intestine, and distal intestine (*n* = 6 *per* sampling day and diet) according to the miRNeasy Tissue/Cells Advanced Mini Kit protocol (Qiagen, Hilden, Germany). RNA was treated with the Turbo DNA-free kit (Invitrogen, Waltham, MA, USA) to avoid genomic DNA contamination. RNA integrity was verified on 1% agarose gel, and RNA concentrations were quantified using a spectrophotometer (Nanodrop® ND1000, Thermo Scientific, Waltham, MA, USA). The cDNA synthesis was performed with 750 ng of total RNA using the SuperScript III reverse transcriptase (Invitrogen, USA) and random primers (Promega, Madison, WI, USA) according to manufacturers’ instructions. Then, for each gene of interest, real-time RT-qPCR was performed using the Lightcycler® 480 II system (Roche Diagnostics, Basel, Switzerland). Primers were designed based on (i) genes identified in a previous study by Frohn et al. (2024) showing that alternative diets based on animal by-product decrease growth performances and are accompanied by changes in the transcriptomic profiles of the liver and the intestine, and (ii) supplemental genes that are key markers of the pathways of interest (Table 2). These genes were involved in immunity and inflammation, structure, coagulation functions, cell protection, antiviral functions, and endoplasmic reticulum stress.

**Table 2:** Primer sequences for RT-qPCR analysis of the liver and proximal and distal intestine

Gene, by category	NCBI accession number	Forward primer (5′-3′)	Reverse primer (3′-5′)	Liver	Proximal intestine	Distal intestine
**Immunity and inflammation**						
* c3*	XM_021561577.2	TGTCACTTCGCCATACACCA	TTTGCAAAACCGTTGGCCC	**x**	**x**	**x**
* prg4*	XM_036968497.1	GGCATAGAAGAGCCAGAACCA	AATGGCATAGGCTGGTCGTC	**x**		
* α2m*	XM_021587033.2	GAGACTGTCCCAAAGGGCTC	CTGCCAACTTCAAGGTGACTTC	**x**		
* il1β*	XM_021590496.2; AJ557021.2	CCCTGCCAGTTGTCTTAGGG	GTCCTTACAGCGCTCCAACT		**x**	**x**
* il6*	NM_001124657.1	CTATCTCTCACTCCTCTCGGC	CTTCCACGGGCTTCTGAAAC		**x**	**x**
* il4*	FN820500.1	GACAATCTTGGCCTCCGTGA	CCACCTGGTCTTGGCTCTTC		**x**	**x**
* tnfα*	AJ278085.1; NM_001124357.1	TGCAAAAGCAGCCATCCATT	AACGAAGAAGAGCCCAGTGT		**x**	**x**
**Structure**						
* tjp1a*	XM_036980662.1	TCAGCTGCGCTATGATGAGG	GGTCGTAACGTAGAGGAGCG		**x**	**x**
* tjp3*	XM_031822609.1	GGGACACAGCCGTGATAGTT	TGACACGCCATTGACCATGA		**x**	**x**
* muc2*	XM_036968565.1; XM_036968563.1	GACCCTGAAGGAGTTCCACG	GTTTGGAGCAGACCTTGGGT		**x**	**x**
* cldn15*	XM_036987534.1; XM_021615039.2	TCTACATTGGCTGGTGCTCG	GTCCTGGCCGTAGGAAGTG		**x**	**x**
* aqp7*	XM_021568669.2	TGAACGTGTTCGAGTGGGAC	AAGGTACTCTCCGAACGCAC		**x**	**x**
* aqp10a*	XM_021621544.2	CGATGTCGTCCAATTGCGG	CAGGTGCCATGCGATGAAGA		**x**	**x**
**Coagulation**						
* serpina10*	XM_036954825.1; XM_036954823.1	TCGCACTTTGTCTCCCCAAA	GCTAGGTTGAGCCCCTTCAG	**x**		
* fbg*	XR_005053092.1; XM_036987044.1	CTGGGTAACGACCGCATCAG	GGCTGTTCCTGAATACCCATC		**x**	**x**
* cd9*	NM_001124324.1	TTCCTGCGTGTAGCGTTGA	AACCCGAACATCGCAAAACC		**x**	**x**
**Cell protection**						
* por*	XM_021623734.2	GTGGTCGCACAATAATGACGTAG	ATGGGGCCACTCACTGTCTG	**x**		
* miox*	XM_021572362.2	CAAGCTGATGCACACCAACC	TCCAGCGATATGACGGCTTC	**x**		
**Cell proliferation**						
* pcna*	XM_036936092.1	ATCCTGAAGTGTGCTGGGAA	TCCCAACTGTTCTACATCGAGA		**x**	**x**
**Antiviral**						
c*h25h*	XM_021564786.2	GGGCTTCTTCTCGTCGGTT	GTCCTCCACAGAAAGCCAGA		**x**	**x**
**Endoplasmic reticulum stress**						
* selenos*	XM_036963928.1; XM_036981274.1	ACTGAAGAGGCCAGCACATC	ACAGGATCCACCTCCCTCTC		**x**	**x**

Real-time RT-qPCR was performed using a reaction mix containing 2 µL of diluted cDNA, 3 µL of Light Cycler 480 SYBR® Green I Master mix (Roche Diagnostics, Switzerland), 0.24 µL of each primer (10X), and 0.52 µL of RNase- and DNase-free water (Thermo Fisher Scientific, USA). Negative controls constituted of RT- and cDNA-free samples. Each RT-qPCR assay was deposited in triplicates on a FrameStar® 384-well skirted qPCR plate (Roche Diagnostics, Switzerland). The qPCR program was initiated at 95 °C for 10 min to denature the cDNA and activate the TAQ polymerase enzyme in a thermocycler (Lightcycler® 480 II Roche thermocycler, Roche Diagnostics, Switzerland). The initiation was followed by 45 amplification cycles, each consisting of successive thermal steps (15 s at 95 °C, 10 s at 60 °C, and 15 s at 72 °C). Melting curves (0.5 °C/10 s from 65 to 95 °C) were run at the end of each amplification cycle to confirm the specificity of the reaction. The relative expression of genes was quantified using the ΔΔCT method (Pfaffl 2001). Elongation factor EF1α (forward 5′-TCCTCTTGGGTTTCGCTG-3′; reverse 3′-ACCCGAGGGACATCCTGTG-5′), 18S rRNA (forward 5′-CGGAGGTTCGAAGACGATCA-3′; reverse 3′-TCGCTAGTTGGCATCGTTTAT-5′), and β-actin (forward 5′-GATGGGCCAGAAAGACAGCTA-3′; reverse 3′-TCGTCCAGTTGACGAT-5′) were employed as non-regulated reference genes in the liver, the proximal intestine, and the distal intestine, respectively. Expression levels were then normalized relative to those of the fasted fish.

### Statistical analysis

Plasma immune markers, histological parameters, and gene expression were statistically analyzed using the Rcmdr package of R software (version 4.1.2).

To assess the early effect of experimental feeds, data from the refeeding period including days 2, 5, and 8 were first treated excluding fasted fish from day 0. Normality of distributions and homogeneity of variances were assessed using a Shapiro–Wilk test and a Levene test, respectively. When these prerequisites were met, the effects of diet, duration of refeeding (days 2, 5, and 8), and the interaction between diet and duration of refeeding (Di:Du) were analyzed using a 2-way analysis of variance (2-way ANOVA), followed by Tukey’s range test.

Then, to assess the effect of short-term fasting followed by refeeding, data including days 0 (fasted fish), 2, 5, and 8 were statistically processed without considering the “diet” factor. As described above, distributions were tested for normality, and variances for homogeneity. When these preconditions were met, the effect of time was assessed using a 1-way analysis of variance (1-way ANOVA) followed by a Tukey’s range test. If one or both conditions were not met, the data were analyzed using the non-parametric Kruskal–Wallis test.

For all analyses, differences were considered statistically significant at the 5% threshold. The *p*-values generated for the two separate statistical analyses (early effect of experimental feeds and effect of short-term fasting followed by refeeding) are shown in separate insets below each graph. Significant differences associated with the early effect of experimental feeds during refeeding are indicated by capital letters. Significant differences associated with the effect of short-term fasting followed by refeeding are indicated by lower-case letters.

## Results

### Plasma immune markers

**Fig. 2:**
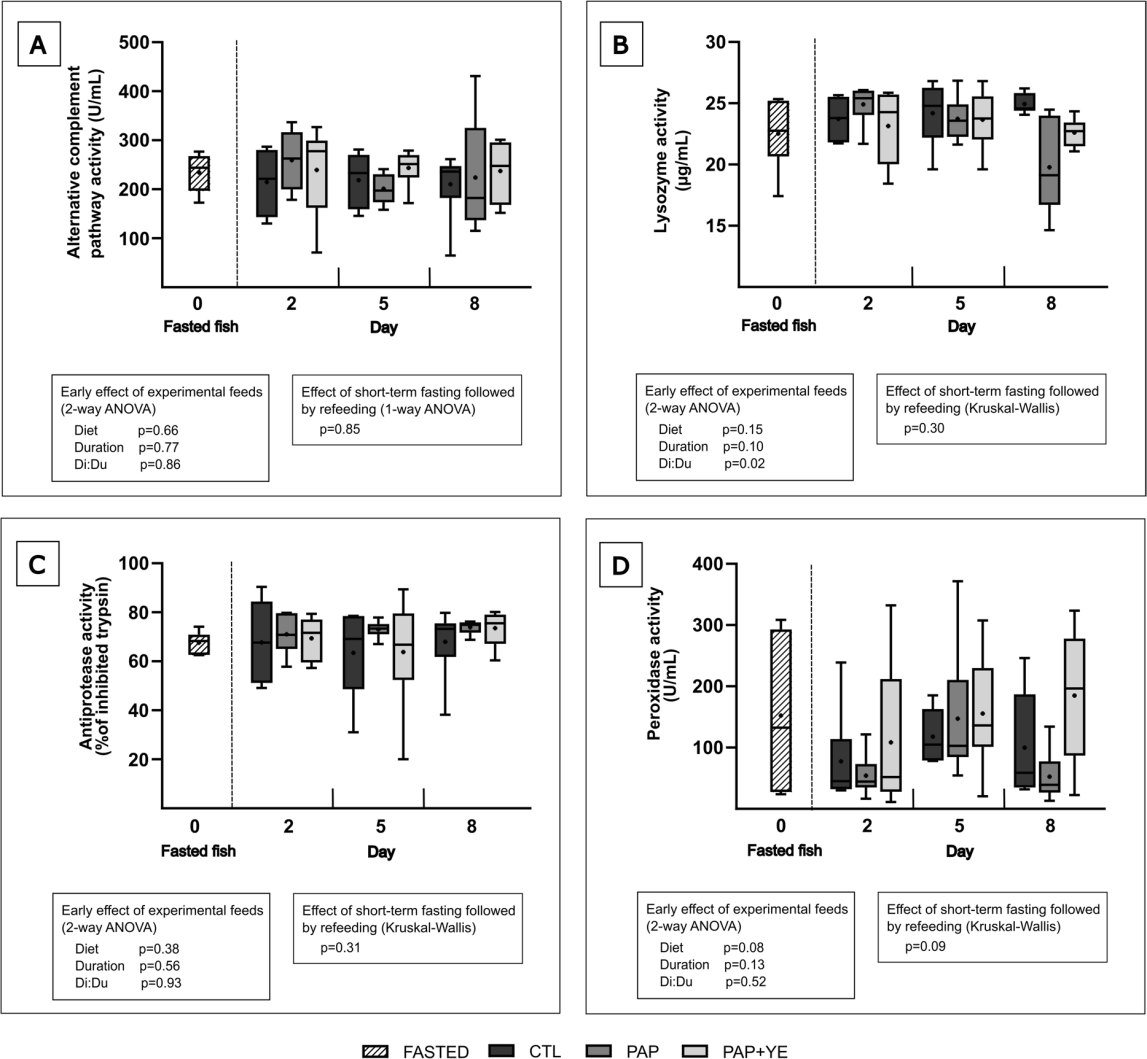
Mean activity (+ standard deviation) (*n* = 6) of alternative complement pathway (**A**), lysozyme activity (**B**), antiprotease activity (**C**), and peroxidase activity (**D**) in the blood plasma of rainbow trout after fasting and on three of the eight subsequent days of refeeding. CTL, commercial-like feed; PAP, terrestrial animal by-products feed; PAP + YE, PAP feed with 3% yeast extract. The *p*-values generated for the two separate statistical analyses (early effect of experimental feeds and effect of short-term fasting followed by refeeding) are shown below each graph

During the refeeding period, neither diet nor duration of refeeding significantly influenced plasma activities of the alternative complement pathway, lysozyme, antiprotease, or peroxidase (2-way ANOVA, *p* > 0.05 for “Diet” and “Duration” factors) (Fig. 2). However, an interaction between diet and refeeding duration (Di:Du) was observed for plasma lysozyme activity, with a significant decrease in fish fed the PAP diet on day 8 compared to day 2, and compared to the fish fed the CTL diet on days 5 and 8 (2-way ANOVA, *p* < 0.05) (Fig. 2B).

No significant effect of short-term fasting followed by refeeding was highlighted for plasma activities of the alternative complement pathway, lysozyme, antiprotease, and peroxidase (1-way ANOVA or Kruskal–Wallis, *p* > 0.05) (Fig. 2).

### Histological parameters of the proximal and distal intestine

**Fig. 3:**
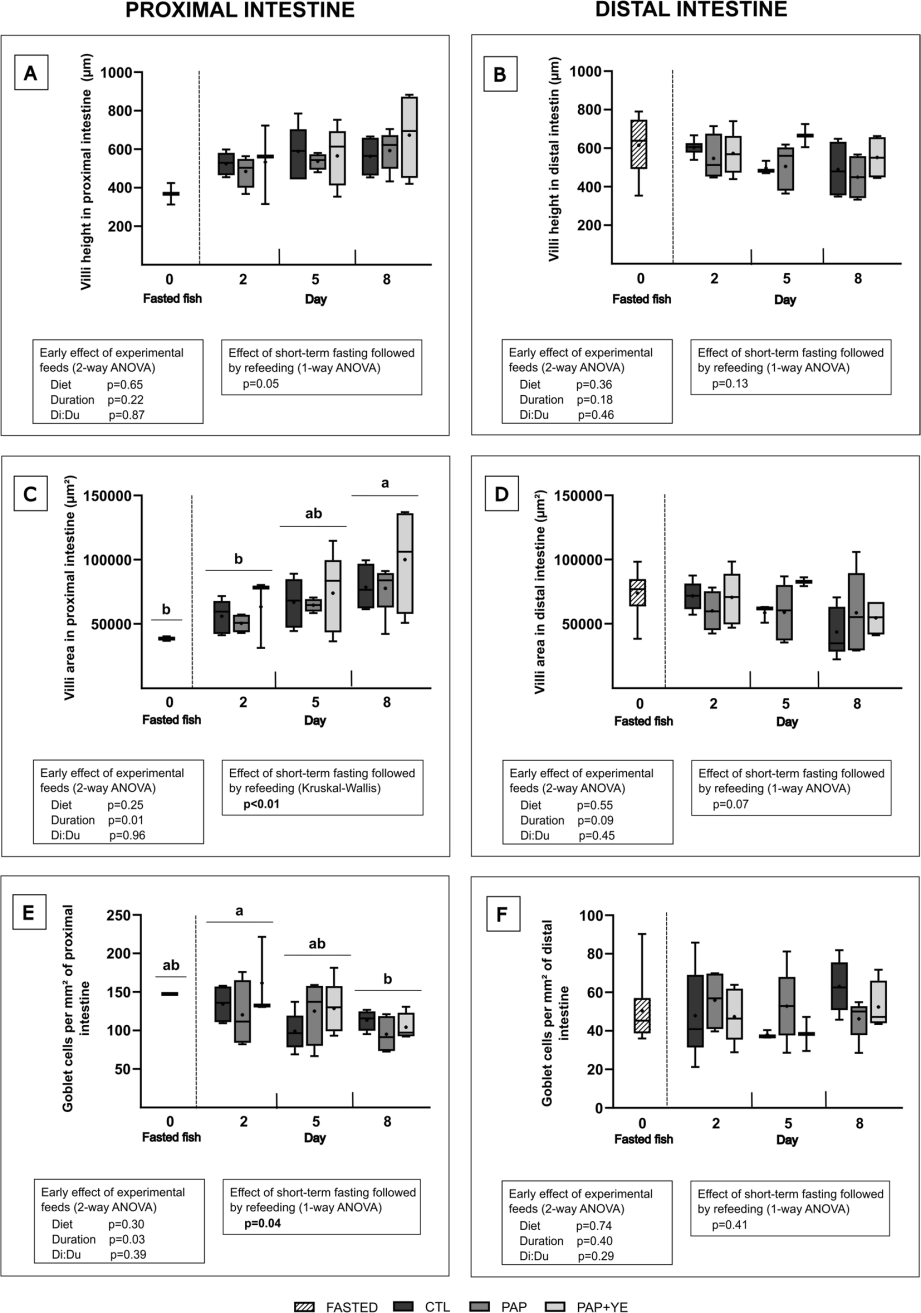
Mean morphometric markers (+ standard deviation) (*n* = 6) of the proximal (**A**, **C**, and **E**) and distal (**B**, **D**, and **F**) intestine of rainbow trout after fasting and on three of the eight subsequent days of refeeding. CTL, commercial-like feed; PAP, terrestrial animal by-products feed; PAP + YE, PAP feed with 3% yeast extract. The *p*-values generated for the two separate statistical analyses (early effect of experimental feeds and effect of short-term fasting followed by refeeding) are shown below each graph. Significant differences associated with the impact of short-term fasting followed by refeeding are indicated by lower-case letters

In the proximal and distal intestine, diet and duration of refeeding had no early effect on villi height (2-way ANOVA, *p* ≥ 0.05 for “Diet” and “Duration” factors) (Fig. 3A and B). Short-term fasting followed by refeeding also had no significant effect on this parameter in either part of the intestine (1-way ANOVA, *p* > 0.05) (Fig. 3A and B).

During refeeding period, in the proximal intestine, the diet did not influence villi area (2-way ANOVA, *p* > 0.05 for “Diet” factor), but refeeding duration did (2-way ANOVA, *p* < 0.05 for “Duration” factor) (Fig. 3C). In the distal intestine, no early effect of diet and duration of refeeding was observed (2-way ANOVA, *p* > 0.05 for “Diet” and “Duration” factors) (Fig. 3D). The intestinal villi area in the proximal part was significantly affected by short-term fasting followed by refeeding, resulting in smaller areas observed in fasted fish and on day 2 of refeeding compared to 8 days of refeeding. Additionally, an intermediate value was observed on the fifth day of refeeding (Kruskal–Wallis, *p* < 0.01) (Fig. 3C). In the distal intestine, short-term fasting followed by refeeding had no significant effect on the villi area (1-way ANOVA, *p* > 0.05) (Fig. 3D).

During refeeding, the diet did not influence the mean density of goblet cells in the proximal and distal intestine (2-way ANOVA, *p* > 0.05 “Diet” factor) (Fig. 3E and F). However, refeeding duration did affect goblet cells density in the proximal intestine (2-way ANOVA, *p* < 0.05 for “Duration” factor) (Fig. 3E) but not in the distal intestine (2-way ANOVA, *p* > 0.05 for “Duration” factor) (Fig. 3F). Short-term fasting followed by refeeding significantly impacted goblet cell density in the proximal part of the intestine, with a significant increase on day 2 of refeeding compared to day 8, and intermediate values observed for fasted fish and on day 5 (1-way ANOVA, *p* < 0.05) (Fig. 3E). Conversely, short-term fasting followed by refeeding had no significant effect on goblet cell density in the distal intestine (1-way ANOVA, *p* > 0.05) (Fig. 3F).

### Cell protection, coagulation, immune, and inflammation-related gene expression in the liver

Expressions of the *por* (NADPH-cytochrome P450 reductase) and *serpina10* (protein Z-dependent protease inhibitor-like) genes, involved in cell protection and coagulation, respectively, were too low to be quantified adequately in the liver (i.e., ≥ 32 amplification cycles (Ct)).

**Fig. 4:**
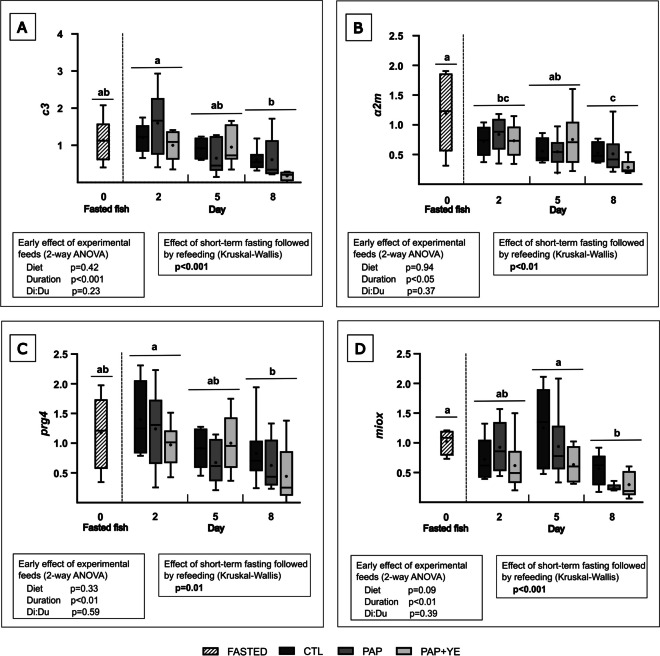
Mean normalized expression (+ standard deviation) (*n* = 6) of *c3* (**A**), *α2m* (**B**), *prg4* (**C**), and *miox* (**D**) genes related to immunity, inflammation, and cell protection in the liver of rainbow trout after fasting and on three of the eight subsequent days of refeeding. CTL, commercial-like feed; PAP, terrestrial animal by-products feed; PAP + YE, PAP feed with 3% yeast extract. *c3*, main complement molecule C3; *prg4*, proteoglycan 4; *miox*, myo-inositol oxygenase; *α2m*, alpha-2-macroglobulin isoform X2. The *p*-values generated for the two separate statistical analyses (early effect of experimental feeds and effect of short-term fasting followed by refeeding) are shown below each graph. Significant differences associated with the impact of short-term fasting followed by refeeding are indicated by lower-case letters

During refeeding, the diets did not significantly influence the expression of genes related to immunity and inflammation (i.e., main complement molecule C3 (*c3*); proteoglycan 4 (*prg4*); and alpha-2-macroglobulin isoform X2 (*α2m*)) (Fig. 4A, B, and C) and to cell protection (i.e., myo-inositol oxygenase (*miox*)) (Fig. 4D) (2-way ANOVA, *p* > 0.05 for “Diet” factor) in the liver. However, their expression was significantly impacted by the duration of refeeding (2-way ANOVA, *p* < 0.05 for “Duration” factor) (Fig. 4A, B, C, and D).

Hepatic expressions of *c3*, *prg4*, *α2m*, and *miox* were significantly influenced by short-term fasting followed by refeeding (Kruskal–Wallis, *p* ⩽ 0.01) (Fig. 4A, B, C, and D). *c3* and *prg4* genes were significantly downregulated on day 8 of refeeding compared to day 2, and their expressions were intermediate in fasted fish and on day 5 (Fig. 4A and C). The expression of the acute-phase protein encoded by *a2m* was significantly upregulated in fasted fish compared to fish refed for 8 days. Intermediate levels of expression were measured on the second and fifth days of refeeding (Fig. 4B). The expression of the *miox* gene peaked in fasted fish and on day 5 of refeeding, was intermediate on day 2, and was significantly downregulated on day 8 (Fig. 4D).

### Gene expression in the proximal and distal intestine

#### Immune, inflammation, and antiviral-related gene expression

In the proximal intestine, expressions of genes related to immunity and inflammation (i.e., *c3*, interleukin-1 beta (*il1β*), interleukin-6 (*il6*), interleukin-4 (*il4*), and tumor necrosis factor alpha (*tnfα*)) and to antiviral functions (i.e., cholesterol 25-hydroxylase gene (*ch25h*)) were too low (Ct ≥ 32) to be quantified adequately using RT-qPCR. In the distal intestine, the expression levels of genes *c3*, apolipoprotein A-II (*apoa2)*, *il4*, *il6*, and *tnfα* were also too low (Ct > 32) to be analyzed.

**Fig. 5:**
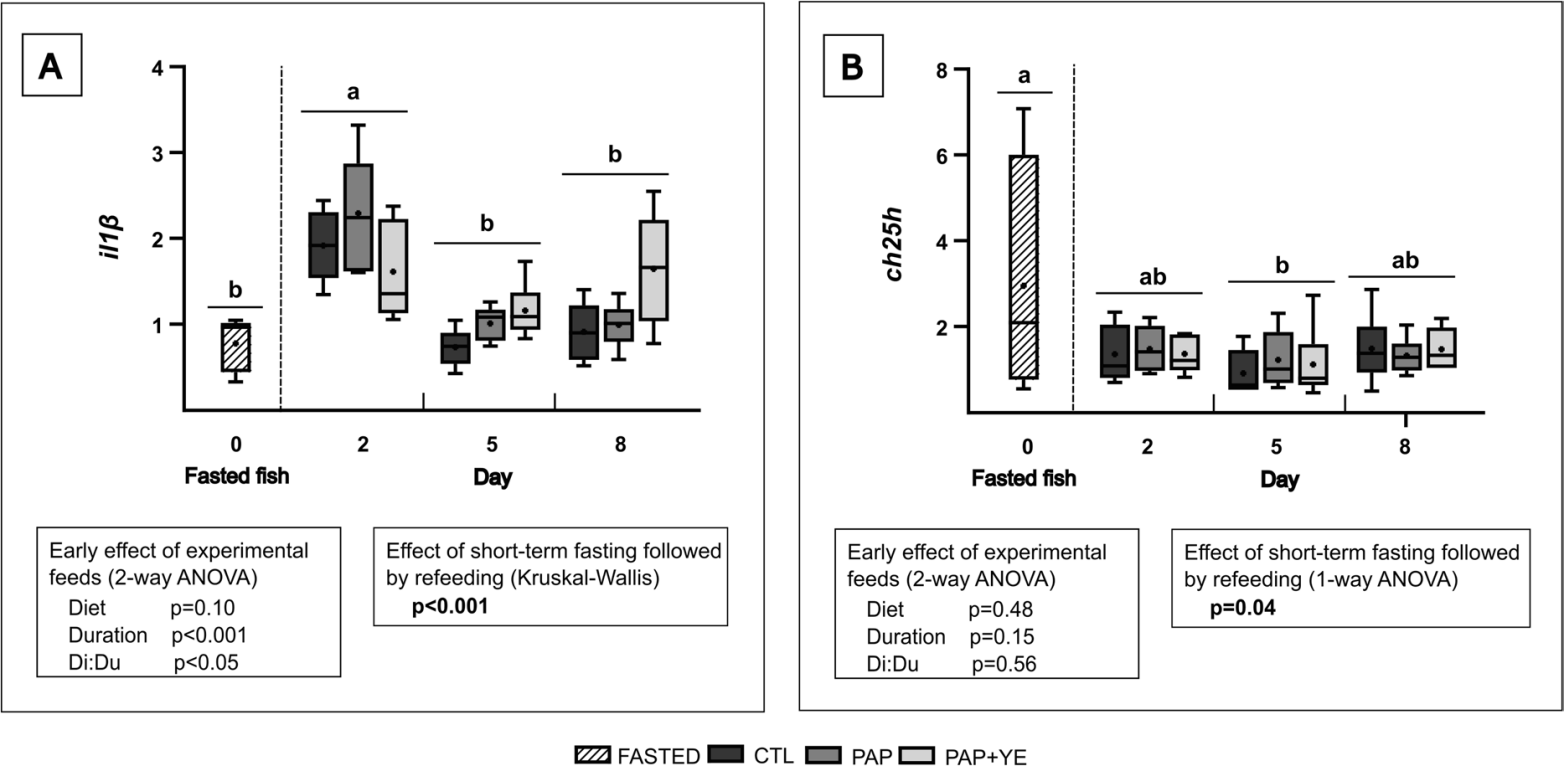
Mean normalized expression (+ standard deviation) (*n* = 6) of *il1β* (**A**) and *ch25h* (**B**) genes related to inflammation and antiviral functions in the distal intestine of rainbow trout after fasting and on three of the eight subsequent days of refeeding. CTL, commercial-like feed; PAP, terrestrial animal by-products feed; PAP + YE, PAP feed with 3% yeast extract; *il1β*, interleukin-1 beta; *ch25h*, cholesterol 25-hydroxylase. The *p*-values generated for the two separate statistical analyses (early effect of experimental feeds and effect of short-term fasting followed by refeeding) are shown below each graph. Significant differences associated with the impact of short-term fasting followed by refeeding are indicated by lower-case letters

In the distal intestine, interleukin *il1β* expression was not impacted by the diets (2-way ANOVA, *p* > 0.05 for “Diet” factor) but refeeding duration did have a significant effect on its expression (2-way ANOVA, *p* < 0.001 for “Duration” factor). Moreover, a significant interaction between diet and duration (Di:Du) due to upregulation of *il1β* on day 2 in fish fed the PAP compared to fish fed the CTL diet on day 5 was highlighted during refeeding (2-way ANOVA, *p* < 0.05) (Fig. 5A). Short-term fasting followed by refeeding also influenced *il1β* expression. Compared to fasted fish, the expression of *il1β* increased after 2 days of refeeding, and then returned to levels similar to those in fasted fish after 5 and 8 days of refeeding (Kruskal–Wallis, *p* < 0.001).

During the period of refeeding, expression of the cholesterol 25-hydroxylase gene (*ch25h*) in the distal intestine was influenced neither by the diet nor by the duration of the feeding period (2-way ANOVA, *p* > 0.05 for “Diet” and “Duration” factors) (Fig. 5B). However, short-term fasting followed by refeeding modulated its expression. On day 5 of refeeding, expression of *ch25h* was significantly downregulated compared to fasted fish and its expression was intermediate on days 2 and 8 (1-way ANOVA, *p* < 0.05) (Fig. 5B).

#### Structure and cell proliferation-related gene expression

In the proximal and distal intestine, expression of tight junction protein-1 *tjp1a* and aquaporins *aqp7* and *aqp10a* were too low to be quantified adequately using RT-qPCR (Ct ≥ 32).

**Fig. 6:**
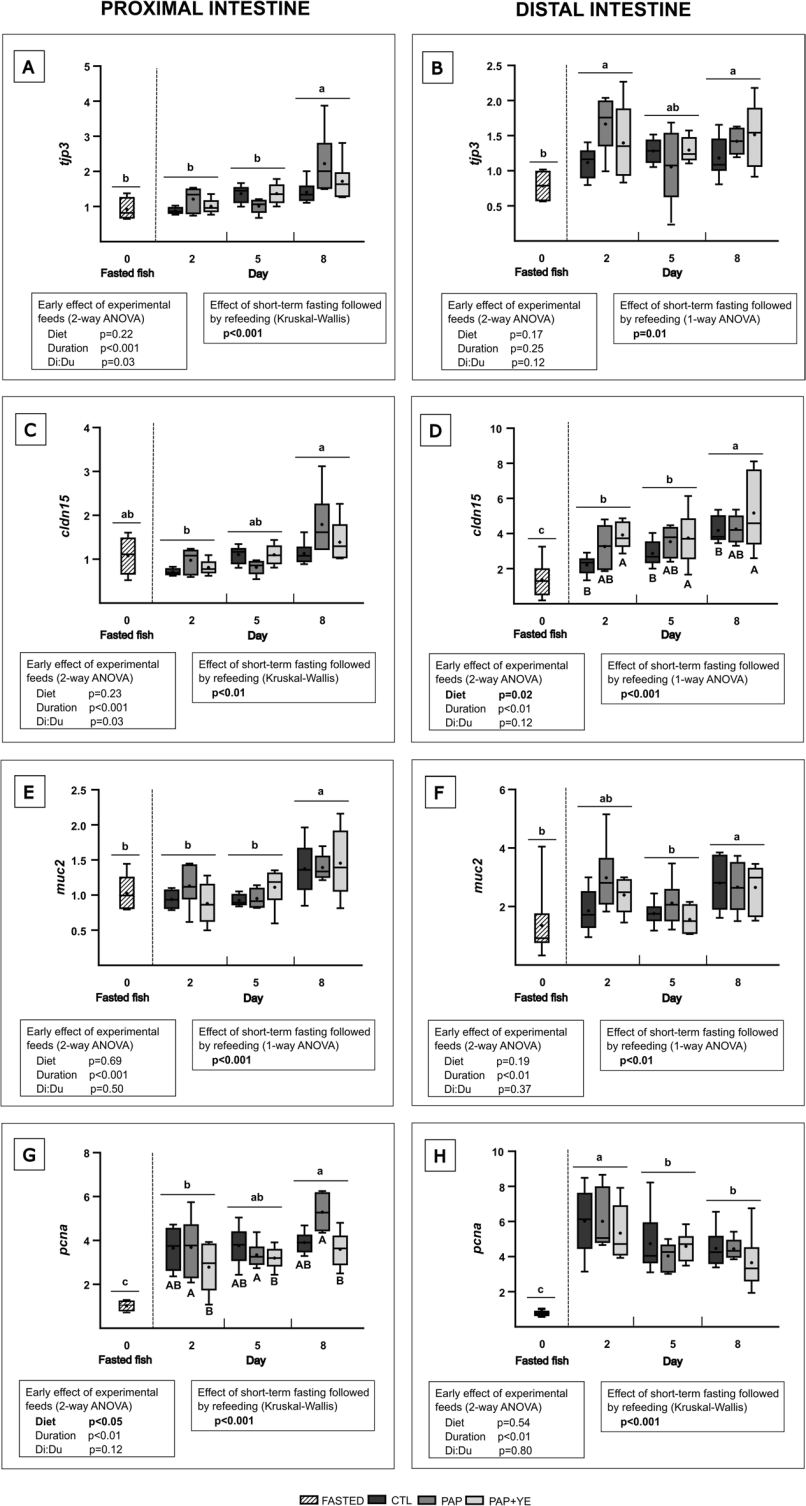
Mean normalized expression (+ standard deviation) (*n* = 6) of genes related to structure, mucus synthesis, and cell proliferation in the proximal (**A**, **C**, **E**, and **G**) and distal (**B**, **D**, **F**, and **H**) parts of the intestine of rainbow trout after fasting and on three of the eight subsequent days of refeeding. CTL, commercial-like feed; PAP, terrestrial animal by-products feed; PAP + YE, PAP feed with 3% yeast extract; *tjp3*, tight junction protein 3; *cldn15*, claudin-15; *muc2*, mucin-2; *pcna*, proliferating cell nuclear antigen. The *p*-values generated for the two separate statistical analyses (early effect of experimental feeds and effect of short-term fasting followed by refeeding) are shown below each graph. Significant differences associated with the early effect of experimental feeds during refeeding are indicated by capital letters. Significant differences associated with the impact of short-term fasting followed by refeeding are indicated by lower-case letters

In the proximal and distal parts of the intestine, expression of the gene encoding the tight junction protein 3 (*tjp3*) was not influenced by the diet (2-way ANOVA, *p* > 0.05 for “Diet” factor) (Fig. 6A and B). In the proximal intestine, *tjp3* expression was influenced by the duration of refeeding (2-way ANOVA, *p* < 0.001 for “Duration” factor) and a significant interaction between diet and duration (Di:Du) was highlighted (2-way ANOVA, *p* < 0.05) (Fig. 6A). In the distal intestine, no significant effect of duration of refeeding was shown (2-way ANOVA, *p* > 0.05 for “Duration” factor) (Fig. 6B). Short-term fasting followed by refeeding had a significant effect on *tjp3* expression in both proximal (Kruskal–Wallis, *p* < 0.001) (Fig. 6A) and distal intestine (1-way ANOVA, *p* < 0.05) (Fig. 6B). In the proximal intestine, *tjp3* was significantly upregulated on day 8 of refeeding compared to fasted fish and to fish refed during 2 or 5 days. In the distal intestine, *tjp3* was significantly upregulated on days 2 and 8 after refeeding compared to fasted fish.

The tight junction protein claudin-15 (*cldn15*) was not early influenced by diet in the proximal intestine during refeeding (2-way ANOVA, *p* > 0.05 for “Diet” factor) (Fig. 6C) but was significantly upregulated in the distal intestine of fish fed the PAP + YE diet compared with those fed the CTL diet after 2, 5, and 8 days of refeeding. Expression of *cldn15* in the distal intestine of fish fed the PAP diet was not significantly different from the two other diets (2-way ANOVA, *p* < 0.05 for “Diet” factor) (Fig. 6D). In the proximal and distal sections, *cldn15* expression was significantly influenced by the duration of refeeding (2-way ANOVA, *p* < 0.01 for “Duration” factor) (Fig. 6C and D), and an interaction between diet and duration (Di:Du) was found in the proximal intestine (2-way ANOVA, *p* < 0.05) (Fig. 6C). In both parts of the intestine, short-term fasting followed by refeeding had a significant effect on *cldn15* expression. In the proximal intestine, *cldn15* was upregulated on day 8 of refeeding compared to day 2 (Kruskal–Wallis, *p* < 0.01) (Fig. 6C). In the distal part, expression of *cldn15* significantly increased between fasted fish and fish refed during 2 and 5 days. A further increase of *cldn15* then occurred between days 5 and 8 of refeeding (1-way ANOVA, *p* < 0.001) (Fig. 6D).

Expression of *muc2* gene, which encodes a protein involved in the secretion of intestinal mucus, was not regulated by diets during refeeding in the proximal and distal parts of the intestine (2-way ANOVA, *p* > 0.05 for “Diet” factor) (Fig. 6E and F). However, its expression was significantly influenced by the duration of the refeeding period in both sections (2-way ANOVA, *p* < 0.01 for “Duration” factor) (Fig. 6E and F). In the proximal intestine, short-term fasting followed by refeeding notably impacted *muc2* gene expression, showing a significant increase between days 5 and 8 days of refeeding (1-way ANOVA, *p* < 0.001) (Fig. 6E). Similarly, in the distal intestine, short-term fasting followed by refeeding led to a significant upregulation of *muc2* on day 8 of refeeding compared to both fasted fish and fish refed for 5 days. However, the expression of *muc2* after 2 days of refeeding did not differ from that measured for fasted fish, and on days 5 and 8 (1-way ANOVA, *p* < 0.01) (Fig. 6F).

The expression of the proliferating cell nuclear antigen (*pcna*) gene, which plays a role in DNA replication and cell proliferation, was early regulated by the diet in the proximal intestine during the refeeding period. It was observed to be lower in fish fed the PAP + YE diet compared to that fed the PAP diet. The expression of *pcna* in the CTL group did not show significant differences compared to the other two diets (2-way ANOVA, *p* < 0.05 for “Diet” factor) (Fig. 6G). In the distal part, no early effect of experimental feeds was observed on *pcna* gene expression (2-way ANOVA, *p* > 0.05 for “Diet” factor) (Fig. 6H). In both proximal and distal parts, the duration of the refeeding period had a significant effect on *pcna* expression (2-way ANOVA, *p* < 0.01 for “Duration” factor) (Fig. 6G and H). In the distal and proximal intestine, *pcna* expression increased after 2 days of refeeding, then rose again between 2 and 8 days of refeeding. The expression level of *pcna* on the fifth day of refeeding was significantly higher than in fasted fish but not different from the expression levels observed in fish refed for 2 or 8 days (Kruskal–Wallis, *p* < 0.001) (Fig. 6G). In the distal part, *pcna* expression was initially lower in fasted fish, then significantly increased on day 2 of refeeding before decreasing on day 5, but its expression levels after 5 and 8 days of refeeding remained nonetheless higher than those of the fasted fish (Kruskal–Wallis, *p* < 0.001) (Fig. 6H).

#### Coagulation-related gene expression

In the proximal and distal intestine, expression of the fibrinogen beta chain (*fbg*) gene was too low (Ct ≥ 32) to be quantified adequately using RT-qPCR (Ct ≥ 32).

**Fig. 7:**
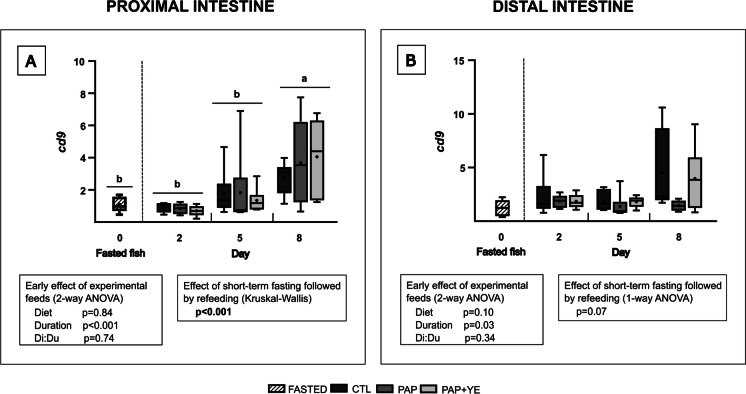
Mean normalized expression (+ standard deviation) (*n* = 6) of the *cd9* gene, involved in coagulation, in the proximal (**A**) and distal (**B**) intestine of rainbow trout after fasting and on three of the eight subsequent days of refeeding. CTL, commercial-like feed; PAP, terrestrial animal by-products feed; PAP + YE, PAP feed with 3% yeast extract; *cd9*, CD9 protein. The *p*-values generated for the two separate statistical analyses (early effect of experimental feeds and effect of short-term fasting followed by refeeding) are shown below each graph. Significant differences associated with the impact of short-term fasting followed by refeeding are indicated by lower-case letters

In both parts of the intestine, *cd9* gene expression was not early affected by experimental feeds (2-way ANOVA, *p* > 0.05 for “Diet” factor) but was significantly modulated by the duration of refeeding (2-way ANOVA, *p* < 0.05 for “Duration” factor) (Fig. 7A and B). A significant effect of short-term fasting followed by refeeding was highlighted in the proximal part of the intestine (Kruskal–Wallis, *p* < 0.001) (Fig. 7A) but not in the distal part (1-way ANOVA, *p* > 0.05) (Fig. 7B). In the proximal intestine, the *cd9* gene was downregulated in fasted fish and on days 2 and 5 of refeeding, compared to day 8.

#### Endoplasmic reticulum stress–related gene expression

**Fig. 8:**
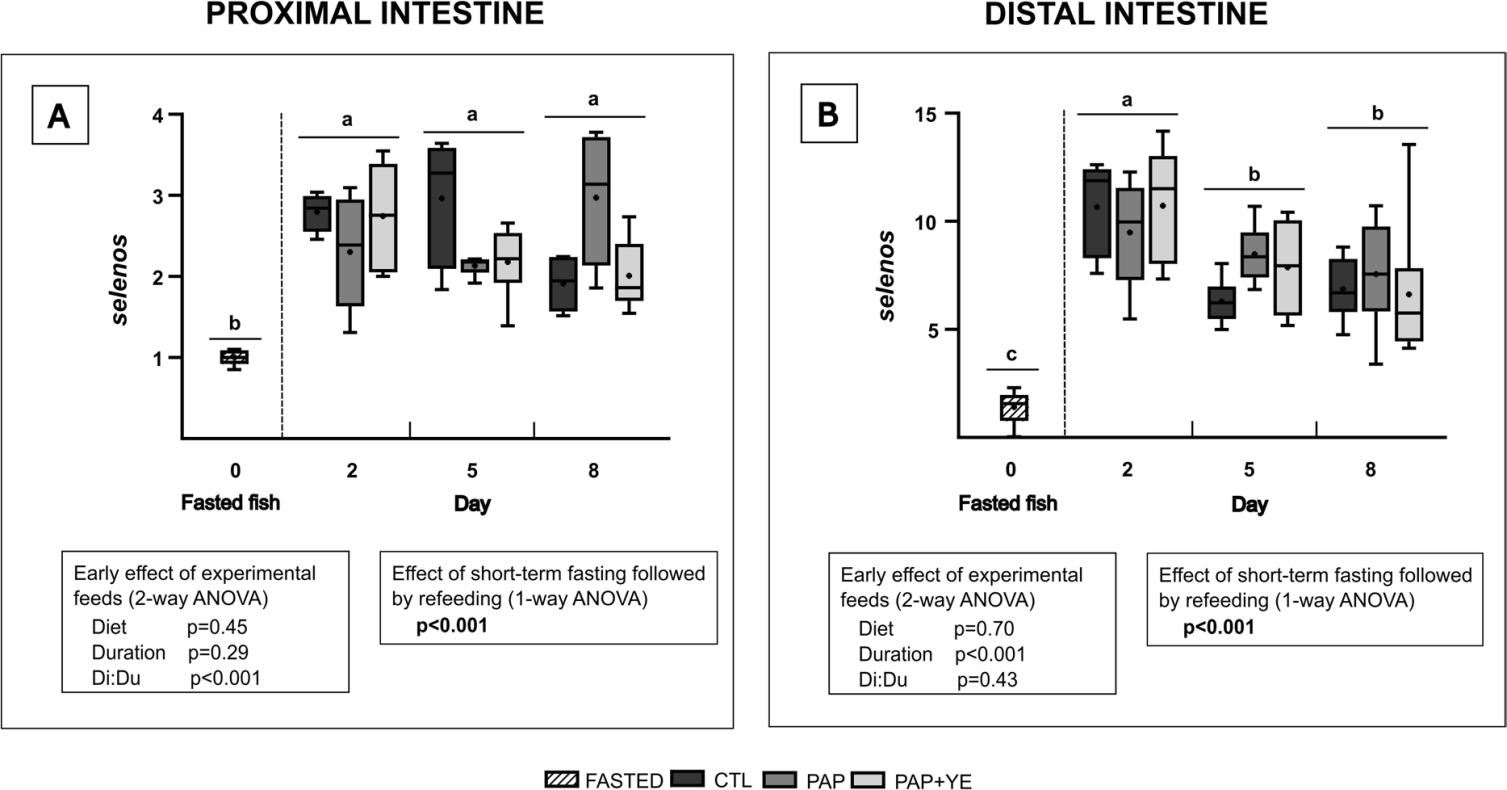
Mean normalized expression (+ standard deviation) (*n* = 6) of the selenoprotein S (*selenos*) gene, involved in the endoplasmic reticulum stress response, in the proximal (**A**) and distal (**B**) intestine of rainbow trout after fasting and on three of the eight subsequent days of refeeding. CTL, commercial-like feed; PAP, terrestrial animal by-products feed; PAP + YE, PAP feed with 3% yeast extract. The *p*-values generated for the two separate statistical analyses (early effect of experimental feeds and effect of short-term fasting followed by refeeding) are shown below each graph. Significant differences associated with the impact of short-term fasting followed by refeeding are indicated by lower-case letters

Expression of selenoprotein S (*selenos*) gene, involved in the endoplasmic reticulum stress response, was not early affected by the diets during refeeding in both parts of the intestine (2-way ANOVA, *p* > 0.05 for “Diet” factor) (Fig. 8A and B). Duration of refeeding period did not impact *selenos* expression in the proximal intestine (2-way ANOVA, *p* > 0.05 for “Duration” factor) (Fig. 8A) but did have a significant effect in the distal intestine (2-way ANOVA, *p* < 0.001 for “Duration” factor) (Fig. 8B). A significant interaction between diet and time (Di:Du) was observed in the proximal intestine (2-way ANOVA, *p* < 0.001). A significant effect of short-term fasting followed by refeeding was highlighted in the proximal and distal intestine. In the proximal section, *selenos* expression was strongly upregulated starting from 2 days of refeeding (1-way ANOVA, *p* < 0.001) (Fig. 8A). Similarly, in the distal part, *selenos* expression strongly increased after 2 days of refeeding, then decreased on days 5 and 8 without returning to the expression level of fasted fish (1-way ANOVA, *p* < 0.001) (Fig. 8B).

## Discussion

The primary aim of this study was to assess the early impact of novel aquafeeds on plasma immune markers, hepatic and intestinal gene expression, and intestinal histology of rainbow trout fed during 8 days with a conventional diet containing 19% fish meal (CTL diet) or two new diets in which the proteins provided by fish meal were replaced with proteins from terrestrial animal by-products, specifically dehydrated poultry proteins, hydrolyzed feather meal proteins, and proteins from pork and poultry blood (PAP and PAP + YE diets). One of the two diets based on animal by-products was supplemented with 3% yeast extract (PAP + YE diet).

As shown in our previous study (Frohn et al. 2024), the use of a diet based on terrestrial animal by-product reduced the growth performances of juvenile rainbow trout, but this could be partially counteracted by adding 3% yeast extract. Over a 12-week feeding period, we have shown that these significant differences in growth performances were accompanied by changes in the transcriptomic profiles of the liver and especially the distal intestine. The modulated genes were related mainly to the innate immune response, inflammation, coagulation, and the response to cellular or pathogenic stress (Frohn et al. 2024). To determine whether the long-term responses observed could be the result of an early response to the diets, we assessed the early effect of these same feeds (CTL, PAP, and PAP + YE diets) on specific plasma immune markers, the expression of genes involved in immunity, inflammation, coagulation, and cell protection in both liver and intestine, and the histology of the intestinal mucosa. Indeed, in a previous study on Atlantic salmon, the first 7 days of feeding with a plant-based diet induced early symptoms of inflammation accompanied by shrinkage of intestinal villi, increased mucus production by goblet cells, and transcriptomic changes for genes related to immune functions (Sahlmann et al. 2013). In our study on rainbow trout, although the fishmeal-free PAP and PAP + YE diets contained 71.09% and 67.89% plant raw materials, respectively, we did not highlight any major early effects of these novel feeds on plasma immune markers, liver and intestinal gene expression, and intestinal histology. Of all the parameters studied, only two intestinal genes were significantly modulated by the diets: *pcna* and *cldn15*. Over the 8 days of refeeding, *pcna* was upregulated from day 2 in the distal intestine of fish fed the PAP diet, which could indicate a rapid resumption of cell proliferation with refeeding. The gene *cldn15* was upregulated in the proximal intestine in response to yeast extract supplementation. These results may suggest that adding yeast as a feed additive in aquafeed could stimulate intestinal cell adhesion after dietary stress, and modulate tissue permeability by preventing the entry of pathogens or toxic molecules (Zhang et al. 2020). Early benefits of including a yeast extract in the diet have been described for common carp (C*yprinus carpio*) (Sakai et al. 2001) and European sea bass (*Dicentrarchus labrax*) (Bagni et al. 2005). However, we were not able to demonstrate an early influence on the immune response or intestinal structure of rainbow trout, as the other selected genes showed no expression or were not significantly impacted by the experimental feeds. Rainbow trout has already shown to be less sensitive than Atlantic salmon to plant-based diets including soybean meal (Refstie et al. 2000). Nevertheless, in the present study, we rather associate the lack of early effects more to the composition of the PAP and PAP + YE diets that were not strictly plant-based. Each of these diets contained 17% of terrestrial animal by-products, ingredients that do not contain anti-nutritional factors (Bureau et al. 1999; Oliva-Teles et al. 2015). Thus, these findings suggest that the long-term effects observed in our previous study by Frohn et al. (2024) were probably more related to chronic adaptation to the new diets than to an early reaction of the fish.

The second objective of this study was to evaluate the effect of a short 4-day fast followed by 8 days of refeeding on the structure of intestinal mucosa, plasma immune markers, and hepatic and intestinal transcriptomic responses. Indeed, intestinal mucosa and the liver are key organs involved in metabolism and maintenance of homeostasis and play a major role in immunity and protective functions. Although fish can survive fasting for several weeks, it can cause early structural changes, induce the production of immune mediators, and modulate transcriptomic responses within the first 2 weeks of feed deprivation (Enyu and Shu-Chien 2011; Secombes et al. 2011; Bar and Volkoff 2012; Mohapatra et al. 2015; Waagbø et al. 2017; Song et al. 2019). In the present study on rainbow trout, 4-day fasting followed by refeeding had no significant impact on plasma immune markers but modulated gene expression in the proximal and distal intestine and affected the structure of the intestinal mucosa.

Depending on the fish species, fasting influences plasma immune markers within the first few days of feed deprivation, and its influence is likely to increase if fasting lasts more than 2 weeks (Pascual et al. 2003; Morales et al. 2004; Feng et al. 2011; Najafi et al. 2014; Eslamloo et al. 2017; Li et al. 2019; Sakyi et al. 2020; Bu et al. 2021). In fish, the complement pathway, which is part of innate immunity and groups together proteins involved in lysis and elimination of pathogens, can be influenced by short- or long-term fasting. For example, tinfoil barb (*Barbonymus schwanenfeldii*) and crucian carp (*Carassius auratus*), exposed to short- (2 weeks) and long-term (60 days) fasting, respectively, showed a significant decrease in plasma alternative complement activity (Eslamloo et al. 2017; Li et al. 2019). Thus, nutrient deprivation could weaken the innate immune system, which in turn could reduce the ability to fight pathogens. In our study on rainbow trout, a decrease in plasma complement pathway activity was not observed, likely due to application of a much shorter fasting period than in the above-mentioned studies. Lysozyme, also part of innate immunity, protects against bacterial infections when released by phagocytes. In the present study, the short fasting period did not seem to influence plasma lysozyme activity in rainbow trout, as it was similar for fasted and refed fish. In the literature, the influence of fasting on plasma lysozyme activity is often described as being dependent on the species studied and the duration of feed deprivation (Bowden 2008). For tinfoil barb, Eslamloo et al. (2017) showed that 1 week of fasting was sufficient to induce an increase in lysozyme activity, which then decreased sharply after 2 weeks of fasting. In Chinese sturgeon (*Acipenser sinensis*), 43 days of fasting clearly induced lysozyme activity (Feng et al. 2011), while prolonged fasting did not influence it in European sea bass, red seabream (*Pagellus bogaraveo*), or European eel (*Anguilla anguilla*) (Caruso et al. 2010). Peroxidase is an antioxidant enzyme involved in eliminating reactive oxygen species released during oxidative stress. In Chinese perch (*Siniperca chuatsi*), for example, fasting can induce such oxidative stress (Bu et al. 2021). Moreover, several studies have demonstrated that plasma peroxidase can be induced soon after fasting begins in fish. For example, in binni (*Mesopotamichthys sharpeyi*) fingerlings, plasma peroxidase activity was strongly induced on the eigth day of fasting (Najafi et al. 2014), while gilthead seabream (*Saprus aurata*) exhibited the highest peroxidase activity after 7 days of fasting (Pascual et al. 2003). However, some studies have shown that if fasting exceeds 2 weeks, peroxidase activity may sharply decrease due to fatigue of the antioxidant system (Morales et al. 2004; Eslamloo et al. 2017). In our study, however, this plasma immune marker was not affected by the 4-day fast followed by refeeding, which raises questions about the duration of fasting required in rainbow trout to induce an increase in peroxidase activity. Plasma antiproteases protect against pathogens by inhibiting the proteases they release. However, the influence of fasting on antiprotease activity remains poorly understood, with existing studies mainly focusing on tissues other than blood or analyzing antiprotease activity after a bacterial challenge (Eslamloo et al. 2017; Esteban et al. 2020; Irungbam et al. 2021). However, in a study involving Nile tilapia (*Oreochromis niloticus*) fasted for 21 days, a duration considerably longer than the 4-day fast experienced by rainbow trout in the current study, plasma antiprotease activity showed a significant increase followed by a decrease during refeeding. However, instead of being directly related to the immune function, these effects were associated to the inhibition of proteases that degrade dietary proteins, thereby preventing them from being digested and assimilated during prolonged fasting (Sakyi et al. 2020). In contrast to what has been described above for other fish species, the short 4-day fast followed by refeeding implemented in our study had no significant impact on plasma alternative complement pathway, lysozyme, antiprotease, or peroxidase activities, suggesting that it may be too short to impact these plasmatic markers in rainbow trout.

Although we did not observe systemic or histological inflammation in response to the 4-day fast, the expression of certain genes related to immunity, inflammation, and cell protection in the liver and intestine differed between fasted and refed fish. In agreement with observations on red porgy (*Pagrus pagrus*), Atlantic salmon (*Salmo salar*), and Japanese grenadier anchovy (*Coilia nasus*) (Martin et al. 2010; Caruso et al. 2012; Wang et al. 2021), hepatic expression of the complement *c3* gene in rainbow trout was highest after 2 days of refeeding. In our study, its expression was not significantly lower in fasted fish, perhaps because the fasting period was shorter than those applied in the three above-mentioned studies which used fasting periods ranging from 14 to 28 days. The protein encoded by the *a2m* gene was upregulated in the liver of fasted trout, proposing early regulation of acute-phase protein homeostasis after a 4-day fast. The opposite was observed in red porgy and Japanese grenadier anchovy, but these fish were subjected to a longer fast, suggesting that prolonged fasting may decrease the activity of this acute-phase protein in fish (Caruso et al. 2012; Wang et al. 2021). To our knowledge, our study is the first report on the influence of fasting on *prg4* expression in fish. *prg4* encodes a proteoglycan that stimulates macrophages during inflammation (Krawetz et al. 2022). In the liver of rainbow trout, its expression decreased between 2 and 8 days of refeeding, even though expression levels during the refeeding phase did not show a statistically significant difference compared to fasting. In the intestine, except for genes encoding cholesterol 25-hydroxylase (*ch25h*) and interleukin-1β (*il1β*), genes related to immune and inflammatory responses were not expressed, or their expression was too low to be adequately quantified in the proximal and distal parts of the intestine. The gene *ch25h* has been related to inflammatory pathologies such as Alzheimer’s disease and atherosclerosis in humans (Liu et al. 2013; Lathe et al. 2014). Additionally, it has been associated to viral infections in Atlantic salmon (Krasnov et al. 2021). In our study, while we observed a higher expression of *ch25h* in fasted fish and a strong but temporary upregulation of *il1β* in response to refeeding, these findings are not sufficient to indicate that short 4-day fast followed by refeeding can induce an immune or inflammatory reaction in the intestine of rainbow trout. In addition, histological analysis of the intestinal mucosa confirmed that there were no signs of inflammation (i.e., increased number of mucus cells and shriveling of the intestinal mucosa). In fact, the intestinal expression levels of genes related to inflammation and immunity in fish appear to be more influenced by long-term fasting period exceeding 2 weeks as it has been previously described for grass carp (*Ctenopharyngodon idellus*), fresh water crayfish marron (*Cherax cainii*), Songpu mirror carp (*Cyprinus carpio L.*), and zebrafish (*Danio rerio*) (Tran et al. 2018; Foysal et al. 2020; Zhao et al. 2022; Jawahar et al. 2022). Regarding coagulation markers in the liver and intestine, although we could not quantify all genes related to this pathway due to low expressions, the downregulation of *cd9* gene in the proximal intestine of fasted fish suggests that fasting could contribute to reducing wound healing and clotting capacities, and that refeeding may restore these functions after 8 days in rainbow trout. Fasting impairs hemostasis and clotting, as observed for traíra (Rios et al. 2005) and for rainbow trout (Salem et al. 2007), in which all transcripts encoding proteins involved in blood coagulation were downregulated in the liver during fasting. Finally, regarding the two selected markers associated with cell protection, only the *miox* gene, which encodes myo-inositol oxygenase, was upregulated in the liver of fasted rainbow trout compared to fish refed for 8 days. In Nile tilapia, myo-inositol is used as an osmolyte to protect cells and maintain normal cytoplasmic osmolarity under hypertonic conditions (Foroutan et al. 2022; Pan et al. 2023). In plants, specifically thale cress (*Arabidopsis thaliana*), *miox* is upregulated under low-nutrient conditions (Alford et al. 2012). Given that fasting can stress cells and impair the osmolarity of rainbow trout (Salem et al. 2007), its upregulation in the liver of fasted fish seems consistent with its protective function. In this way, its downregulation after 8 days of refeeding could therefore be related to the presence of nutrients in the gut. In the intestine, especially in the distal section, the expression of *selenos*, a gene encoding a protein involved in protein folding in the endoplasmic reticulum and in glucose metabolism, was also strongly influenced by the transition from fasting to refeeding by being significantly upregulated in refed fish. These results align with previous descriptions in mammals, indicating that selenoprotein S expression is regulated by glucose deprivation (Gao et al. 2004; Schröder and Kaufman 2005). In fish, feed deprivation is also likely to induce endoplasmic reticulum stress and consequently a protein folding defect. Indeed, a study on Masu salmon (*Oncorhynchus masou masou*) showed that the transition from a 3-day fast to refeeding was accompanied by an overexpression of genes involved in protein folding in the first 24 h post-refeeding (Kondo et al. 2020). Overall, although not all markers considered in this study were influenced by the transition from 4-day fasting to refeeding, our results still suggest that short-term fasting is likely to influence the expression of several genes involved in immune and inflammatory functions, coagulation, cell protection, and endoplasmic reticulum stress. Even though fish are known to be capable of enduring fasts lasting several days, these gene expression variations suggest significant physiological adaptations of the animal in response to fasting.

Indeed, during fasting in fish, structural changes include atrophy of the digestive tract and a decrease in the length of the gut, followed by intestinal weight loss and a decrease in the surface area of the intestinal mucosa. A decrease in gut length and mass has been observed in neon damselfish (*Pomacentrus coelestis*) subjected to 16 days of fasting, in javelin goby (*Synechogobius hasta*) after 7 days of fasting (Hall and Bellwood 1995; Zhou et al. 2022), and in common carp (*Cyprinus carpi*) subjected to 6 months of starvation (Gas and Noailliac-Depeyre 1976). As shown for traíra (*Hoplias malabaricus*), a decrease in gut length can also be accompanied by a decrease in the height of intestinal mucosa and the villi surface area (Rios et al. 2004). In the present study, short-term fasting followed by refeeding had no significant effect on intestinal villi height in the proximal or distal intestine of the rainbow trout. However, villi surface area in the proximal intestine significantly increased after 8 days of refeeding compared to fasted fish, which indicates a strong relation between refeeding and absorptive capacity. This increase in villi surface area is also consistent with the upregulation of *pcna* in the proximal intestine of rainbow trout during refeeding, results that were also observed in the intestine of frogs (*Xenopus laevis*) refed after fasting (Tamaoki et al. 2016). Although *pcna* expression was similarly regulated in the proximal and distal parts in response to refeeding, no significant increase in villi surface area was observed in the distal intestine. This shift is certainly associated to the transit of feed and nutrients through the gut, which reaches the distal part last. However, our results are consistent with previous observations made in salmonids. Indeed, for short- or medium-term fasting, the gut responds rapidly to fasting, and the effects seem to be reversible upon refeeding, as described for rainbow trout and Atlantic salmon being respectively fasted for 3 weeks and 40 days (Weatherley and Gill 1981; Krogdahl and Marie Bakke-McKellep 2005). Regarding the expression of genes involved in enterocyte adhesion and intestinal permeability, *tjp3* and *cldn15* were modulated by the transition from fasting to refeeding, which provides further evidence of the influence of short-term fasting (< 2 weeks) on gut structure. However, the response of genes encoding tight junction proteins differed between the sections of the intestine: the distal section seemed to respond earlier to refeeding, which could indicate its higher sensitivity to dietary changes (Van Den Ingh et al. 1991; Baeverfjord and Krogdahl 1996). Like tight junction proteins, goblet cells play an important role in maintaining intestinal homeostasis since they produce intestinal mucus and are present throughout the digestive tract. As a lubricant, mucus facilitates the passage of feed and nutrients through the gut and protects the intestinal mucosa (Khojasteh 2012). In our study, although short-term fasting did not significantly influence the density of mucus cells in the distal intestine of rainbow trout, the number of goblet cells increased after 2 days of refeeding in the proximal intestine, probably in response to the presence of feed in the digestive tract. Conversely, the modulation observed for *muc2* gene expression (i.e., low in fasted fish but higher in both sections of the intestine after 8 days of refeeding) supports the observations made for northern pike (*Esox lucius*) and southern catfish (*Silurus meridionalis*) showing that mucus cell dynamic can vary depending on whether the fish is fed or not (Bucke 1971; Zeng et al. 2012). Overall, although we observed shifted expression dynamics in some gene expressions between the two parts of the intestine, our results indicate that even a short 4-day fast followed by refeeding can impact transcriptomic markers involved in structure, cell adhesion, and proliferation and mucus production. Furthermore, the short fast followed by refeeding appears to have more influenced the villi surface area and the density of goblet cells in the proximal intestine, but we assume that similar effects might have been observed in the distal part if the duration of fasting had been longer than 4 days.

In conclusion, diets based on terrestrial by-products supplemented or not with yeast extract did not have an early major impact on blood plasma immunity, hepatic and intestinal specific gene expression, and intestinal histology of rainbow trout during 8 days of feeding. Thus, the reduction in growth performance associated to the PAP diet, the improvement of this diet with the addition of yeast extract, and the changes in hepatic and intestinal transcriptomic profile observed during 12 weeks of feeding (Frohn et al. 2024) may not be related to an early response, but rather to long-term adaptation of fish. Nevertheless, our study demonstrated that short-term fasting can have a significant impact on rainbow trout physiology. Although there was no impact on plasma immune markers, the 4-day fast followed by refeeding impacted intestinal histology and modulated the expression of certain genes related to inflammation and immunity, coagulation, structure, cell protection, and endoplasmic reticulum stress, in the liver and intestine. Given that these periods of short fasting are often applied in fish farms before animal handling, to increase survival, maintain growth, or improve the immune defenses of fish in situations of stress or epizootic disease, the effects highlighted in this study will need to be examined in greater depth in future studies, to confirm the real benefits or disadvantages of this type of practice.

### Supplementary Information

Below is the link to the electronic supplementary material.

Supplementary file1 (DOCX 225 KB)

## Data Availability

The data that support the findings of this study are available from the corresponding author upon reasonable request.
