# Supplementary material for: Gut physiology of rainbow trout (Oncorhynchus mykiss) is influenced more by short-term fasting followed by refeeding than by feeding fishmeal-free diets
Source: Fish Physiol Biochem. 2024 Apr 16;50(3):1281–303. doi: 10.1007/s10695-024-01339-0 (PMC11213814; doi:10.1007/s10695-024-01339-0)
Supplement: Supplementary file 1 — Supplementary file1 (DOCX 225 KB) [file 10695_2024_1339_MOESM1_ESM.docx]

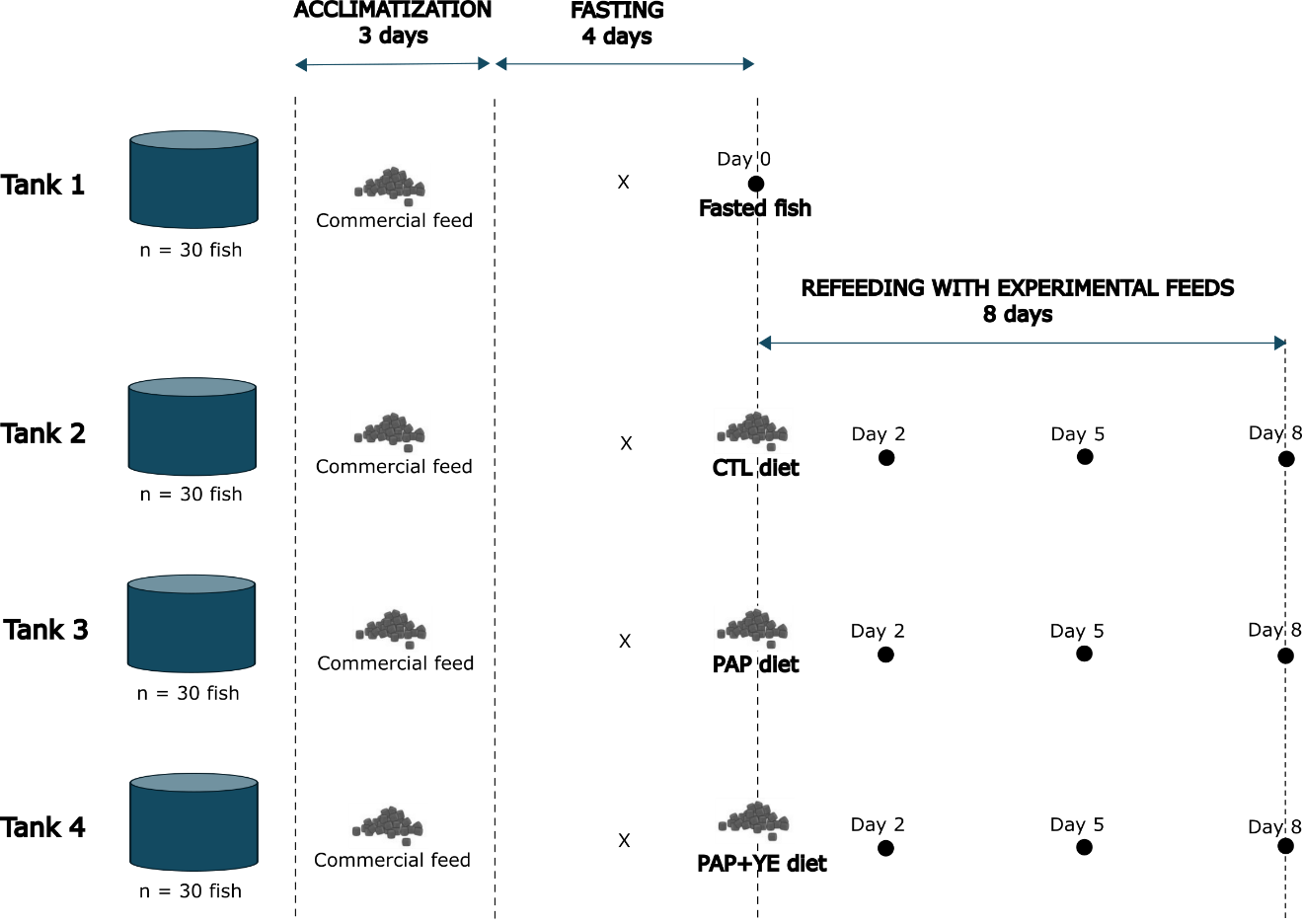


**Supplementary Figure 1.** Schematic diagram of the experimental protocol. CTL, commercial-like feed; PAP, terrestrial animal by-products feed; PAP+YE, PAP feed with 3% yeast extract. Points in bold (⦁) indicate sampling days associated to each tank.
